# The Use of Machine Learning to Estimate Ground Reaction Forces During Running: A Scoping Review of the Current Practices

**DOI:** 10.3390/s26082502

**Published:** 2026-04-18

**Authors:** Anderson Souza Oliveira, Morteza Yaserifar, Cristina-Ioana Pîrșcoveanu

**Affiliations:** 1 Department of Materials and Production, Aalborg University, 9220 Aalborg, Denmark; 2 Department of Exercise Physiology, University of Mazandaran, Babolsar 47415, Iran; yaserifm@myumanitoba.ca; 3 Department of Health Science and Technology, Aalborg University, 9260 Aalborg, Denmark; civ@hst.aau.dk

**Keywords:** ground reaction forces, wearable technology, machine learning, deep learning, running biomechanics

## Abstract

Ground reaction forces (GRFs) are essential for assessing running biomechanics, and the combination of wearable sensors and machine learning offers an accessible alternative for estimating GRFs outside controlled environments. This scoping review summarized current methods used to predict GRFs during running. A structured search (2019–2025) identified 36 studies, from which 37% did not report participant’s training status, and 59% of all participants were males. Treadmill running was assessed in 58% of studies, which included larger samples (median N = 28) and more steps/participant (median = 65) than overground studies (median N = 14; median = 32). Deep learning models, particularly LSTM and Bi-LSTM networks, were the most applied techniques, though presenting similar accuracies compared to classical regression methods. Vertical GRF predictions were the most accurate, while mediolateral GRF predictions remain challenging. GRF-derived variables such as peak forces, impact peaks, and impulses were predicted more accurately than region-dependent metrics like loading rates. Notably, no study validated treadmill-trained models on overground running, limiting real-world generalizability. Future work should prioritize larger, sex-balanced cohorts, improving prediction of mediolateral GRFs and loading rates, and explore validating treadmill-based models in overground conditions. In conclusion, although machine learning shows promise for GRF predictions, key methodological gaps must be addressed to enable robust, real-world applications.

## 1. Introduction

Running is a highly accessible physical activity as it requires minimal equipment and can be performed in any environmental conditions. Recreational and elite athletes perform running to improve their fitness levels, while sedentary people and sports enthusiasts find running an alternative to exercise for weight loss and health maintenance. Understanding the relevance of the forces applied to the floor during running has relevant implications for sports performance and sports medicine. Regarding performance, assessing the vertical ground reaction force (GRF) provides estimates of the stance and swing time and estimates braking and propulsion forces, as well as the peak forces generated during running. Regarding injury prevention, vertical forces provide estimates on the early impact forces, investigated using the rate of increase in force (e.g., loading rate) following initial contact [1,2]. Moreover, vertical ground reaction forces can be used to determine running techniques and differentiate between rearfoot and forefoot strikes [3,4]. Therefore, assessing vertical ground reaction forces can provide practical information for runners, coaches, researchers, and industry-oriented businesses.

Despite the high relevance, assessments of ground reaction force are limited. The state-of-the-art method is piezoelectric force platforms that measure tri-dimensional forces. However, this method is expensive and limits data acquisition due to the stationary nature of the equipment. A secondary option is the use of plantar pressure insoles that allow measurement of vertical forces in highly mobile conditions [5]. Such insoles provide reasonable data quality unconstrained regarding placement, as wireless methods allow the user to maintain a more natural running pattern independent of the running environment. The downside of this technology is the price and the durability of the sensors, making it difficult to popularize the method amongst athletes and coaches. Therefore, current direct measurements of ground reaction forces are limited and are mostly impractical for assessing real-world performance during training/competitions.

Sensor miniaturization has played a crucial role in changing the way researchers investigate running mechanics. Inertial measurement units (IMUs) have become a favorite method to assess human motion and opened possibilities to acquire motion data outdoors. There is a growing interest in using wearable sensors to investigate running mechanics and variables related to ground reaction forces, such as contact times and impact properties. The popularization of machine learning techniques has unlocked the potential of using data from simplified sensors to estimate the highly valuable but less accessible ground reaction forces. Several studies have been published within the last 20 years on the topic, and a first literature review regarding the methods used to estimate GRFs has been published in 2018 [6], which included nine studies related to running. In 2022, another literature review regarding the use of machine learning in running biomechanics evaluated seven studies predicting GRF or GRF-related variables [7]. While the 2022 review addressed machine learning applications in running biomechanics more broadly, only a limited number of included studies specifically focused on GRF prediction, whereas the 2018 review remained centered on GRF estimation methods.

From the publication of Ancillao et al. [6], several new studies have been published using different recording devices and methods, as well as different machine learning algorithms that are becoming ever faster and more accurate. These mathematical models are becoming more influential in sports sciences, including artificial neural networks, support vector machines, decision trees, binary logistic regressions, and random forests, and are used on a wide variety of topics from injury prevention to prediction of match outcomes [8,9]. Therefore, it seems like a timely moment to scope the literature and present the current state of advances and propose future directions towards improving research on estimating ground reaction forces during running.

## 2. Materials and Methods

### 2.1. Search Strategy

This review follows the suggestions of Munn et al. [10] for scoping reviews, where we seek to identify new research practices and potential gaps in the most recent literature. The completed PRISMA-ScR-Fillable checklist is provided in the Supplementary Materials. We considered scientific manuscripts written in English which investigated the feasibility of estimating GRF during running using a wide variety of data acquisition methods and a variety of machine learning methods. The publication date was limited from 2019 to the date of the electronic literature search on 15 December 2025. Scientific studies were searched in four different databases (Web of Science, ScienceDirect, Scopus, and PubMed). Two researchers performed the search using a search strategy employing Boolean operators (AND, OR) to combine keywords related to machine learning (e.g., “artificial intelligence”, “machine learning”, “deep learning”, “neural network”, “SVM”, “support vector machine”, “KNN”, “k-nearest neighbors”, “trees”, “decision trees”, “linear regression”, “predicting”, “clustering”, “PCA”, “principal component analysis”), running activity (e.g., “running”, “jogging”, “sprinting”), and force/biomechanical measurements (e.g., “force”, “ground reaction force”, “GRF”, “plantar pressure”, “insoles”, “pressure insoles”, “motion capture”, “optical motion capture”, “reflective markers”, “wearable sensors”, “inertial motion capture”, “inertial measurement units”, “acceleration”). Database-specific syntax was employed where necessary, with Medical Subject Heading (MeSH) terms incorporated for PubMed searches.

### 2.2. Eligibility Criteria

We used terms seeking to identify studies involving running, ground reaction forces, data acquisition of human movement, and applied machine learning. In total, 600 studies were identified. After removing duplicates, 237 unique records were screened, resulting in 146 title-and-abstract assessments and 63 exclusions. Of the 87 full-text articles reviewed, 51 were excluded for reasons such as lacking machine-learning methods, not investigating GRF, using outdated or non-original data, or not involving prediction or running contexts. Studies were additionally required to (1) investigate treadmill or overground running; (2) use mechanical instrumentation such as IMUs, reflective markers, or pressure insoles; (3) report GRF time series or GRF-derived variables; and (4) apply a machine-learning model such as linear regression or neural networks for GRF estimation. By following the established criteria, only 36 studies met the inclusion criteria and were included in our results.

**Figure 1:**
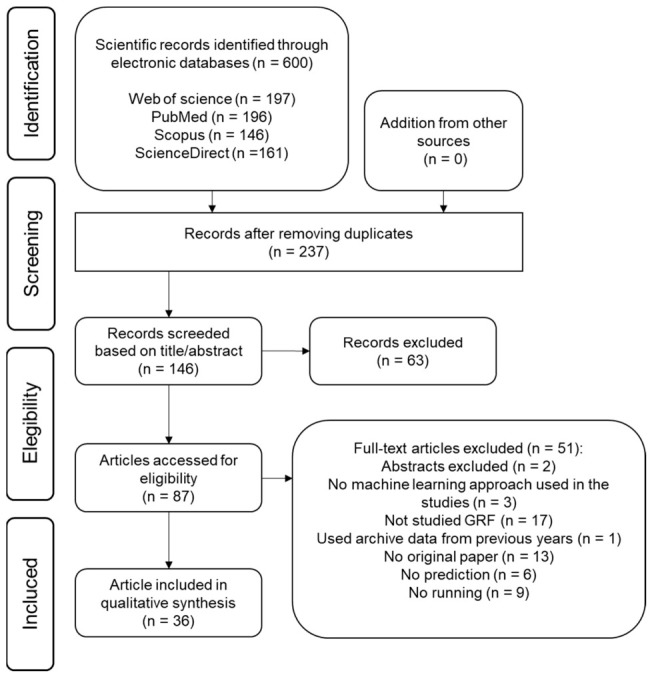
Flowchart of the systematic screening process (PRISMA).

Study selection followed a structured multi-stage process to ensure reliability and repeatability. Initial screening of titles and abstracts was performed by one primary screener. To minimize bias and ensure accuracy, any studies flagged as uncertain or where eligibility was unclear were subjected to independent review by two additional researchers. Disagreements regarding study inclusion were resolved through a collaborative consensus-based discussion involving all three researchers. This tiered approach ensured that all final inclusions met the pre-defined criteria via team validation. The flow diagram of our manuscript search and screen process is shown in Figure 1.

## 3. Results

### 3.1. Study Demographics

**Figure 2:**
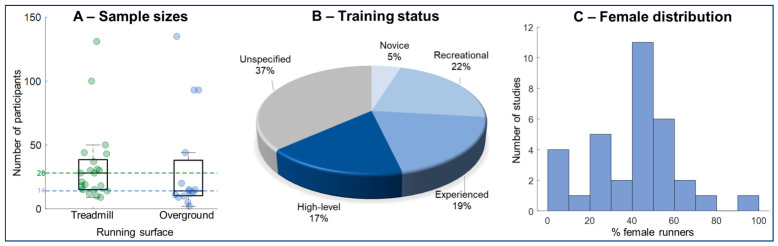
Distributions of sample size (**A**), training status (**B**), and the percentage of female runners in the study samples (**C**). **In (A), the horizontal dashed lines represent the median values for the sample of the same color.**

In general, the 36 studies assessed running from 1207 participants, with a median of 18 participants per study (mean = 33 ± 34 participants). Regarding age, participants presented a mean age of 27 ± 5 years (Table 1). When studies are separated into treadmill and overground classes, treadmill studies assessed a median of 28 participants (mean ± SD: 35 ± 30 participants) compared to a median of only 14 participants in overground studies (mean ± SD: 33 ± 40 participants, Figure 2A).

Training status of the study participants has been classified within four categories: (1) sedentary or minimally trained individuals (novice) that do not perform systematic running training; (2) recreational runners that practice running with systematic training regimen; (3) experienced runners that present >5 years running experience and follow systematic training regimen, but do not compete in high-level competitions; and (4) high-level runners that perform semi-professionally or professionally. We found that four studies [14,18,23,33] acquired data from different groups, leading to 41 group entries across the 36 studies investigated. It was found that 27% of studies assessed either novice or recreational runners, whereas 36% of studies assessed experienced or professional runners (Figure 2B). Moreover, 15 studies (37% of the total) did not report on the training status of their participants.

Sex distribution has been reported by 31 studies, demonstrating an imbalanced sex distribution across studies. We found that 59% of all participants are males and 41% are females. In terms of sample distribution within studies, 16 out of the 31 studies (~52% of studies) have equal or fewer than 40% females in the sample (Figure 2C), whereas only 30% of studies have more than 50% females in the sample.

### 3.2. Running Tasks, Speed Selection and Number of Steps

From the 36 studies, 21 investigated treadmill running (~58% of the total), while 15 studies (42%) investigated overground running. Two studies [24,25] investigated uphill/downhill running, while Yilmazgün et al. [45] investigated running on a flat surface, as well as running spin turn and running step turn. All other studies investigated running on flat surfaces.

**Figure 3:**
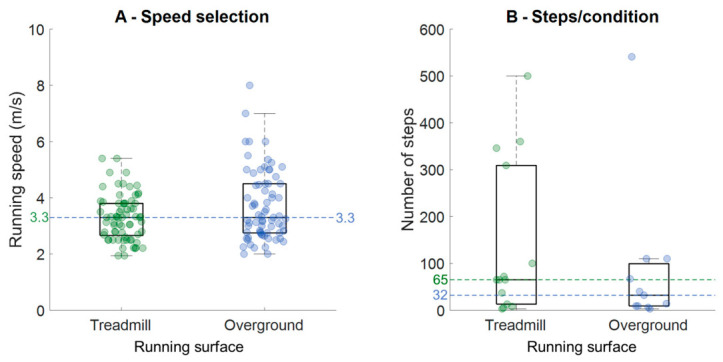
Boxplots representing the (**A**) assessed running speeds (**B**) and the number of steps acquired per participant in the different running speeds within studies evaluating treadmill running (green circles) and overground running (blue circles). **In (A), the horizontal dashed lines represent the median values for the sample of the same color.**

The running speeds investigated across the studies ranged from 1.94 m/s to 8.0 m/s (median: 3.3 m/s; mean: 3.5 ± 1.1 m/s, Figure 3A, Table 1), while the majority of studies investigated speeds between 2.4 and 4.0 m/s. Johnson et al. [23] evaluated different fixed speeds (slow, moderate, and fast) as well as acceleration and deceleration running, while Pogson et al. [17] evaluated high-speed overground running above 6 m/s. One study did not report on the running speeds investigated [34]. Across all studies, seven (~19%) investigated only one running speed that sometimes was the preferred running speed [11,14,26,27,29,39,46], while ~19% of studies evaluated more than five speeds [17,18,31,32,37,38,41].

Regarding the number of steps used for testing/training machine learning algorithms, it is noteworthy that studies named this variable as strides (ipsilateral measures), steps, footfalls, data samples, or events in their methods, and we are calling it steps for consistency. We found that treadmill-based studies acquired data from ~496 steps per participant on average, whereas overground-based studies acquired data from ~710 steps per participant. However, these numbers are skewed by studies recording ≥800 steps/participant on treadmills [15,16,18,25,41], whereas overground running was highly influenced by Donahue and Hahn [31], who recorded more than 7000 steps/participant. Another relevant exception is the study of Johnson et al. [23], who reported the use of 19,400 events for training their algorithms, but the sample size was not reported. The authors reported a total of 250 predicted values from a five-participant testing sample, but these numbers were not included in the calculations, as there was no report of the sample size from the training dataset. When the number of running speeds was accounted for to determine the number of steps per participant, treadmill-based studies assessed a median of 65 steps (mean: 139 ± 165 steps) and overground-based studies assessed a median of 32 steps (mean: 86 ± 156 steps, Figure 3B, Table 1).

### 3.3. Ground Truth and Alternative Instrumentations

The majority of studies investigating treadmill running used instrumented treadmills to assess ground truth GRFs, except for Tedesco et al. [22], who used shoe insoles (Table 1). Eleven out of the 15 studies assessing overground walking (73%) assessed ground truth GRF using force plates, whereas four studies used shoe insoles [19,31,34].

Regarding alternative methods for GRF estimation, the most used method was inertial motion capture (22 studies, ~60% of total). Five studies estimated GRF using a single IMU placed on the trunk/sacrum [17,24,30,41,44] or shank [44], while the other studies used from two to five IMUs. Two studies [43,46] did not report the number of IMUs used in their analyses. The IMU data were typically treated as time-series and mapped to GRF/GRF-derived targets using deep sequence models or neural regressors (e.g., [16,18,31,41,42,43]).

There were five studies estimating GRF or GRF-based variables from optical motion capture [11,12,14,28,36]. Retro-reflexive markers were predominantly placed on the lower limbs. Some studies assessed alternative technologies, such as 2D video analyses [29,33], in-shoe pressure sensors [25,39], and high-performance microphones to acquire the sound of footsteps during running [26]. Two studies combined technologies, such as instrumented shoes/insoles and IMUs [15,40], GPS-aided inertial navigation system [19], and a combination of IMUs with optical markers [46].

### 3.4. Machine Learning Algorithm Selection

**Figure 4:**
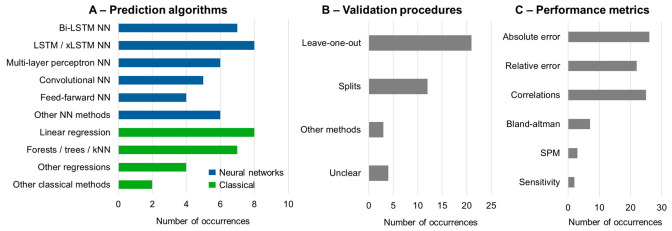
(**A**) Occurrence of machine learning algorithms used for estimating ground reaction forces across all assessed studies. Prediction models based on neural networks (NN) are illustrated in blue, whereas classic algorithms are illustrated in green. In (**B**), the occurrence of the different types of validation procedures. In (**C**), the occurrence of different performance metric used to assess the prediction qualities. NN: neural network; LSTM: long-short term memory; Bi-LSTM: bilateral long-short term memory; xLSTM: extended long-short term memory; kNN: K-Nearest Neighbors; SPM: statistical parametric mapping.

Across the included studies, algorithms based on deep learning were the most used (Figure 4A, Table 2). In particular, 6 out of 36 studies have applied regular long-short term memory neural networks (LSTM, [19,28,31,34,43,46]), and 7 out of 36 studies applied bi-directional Bi-LSTM [21,24,25,31,33,40,42]. Convolutional neural networks (CNNs) were used by 5 out of 36 studies [14,16,18,23,45], while 6 out of 36 studies relied on multi-layer perceptrons (MLPs) [12,17,18,22,35,44] and 4 out of 36 studies used feed-forward neural networks (FFNNs) [12,22,35,44] either as primary estimators or as part of hybrid pipelines with multiple algorithms.

Classical machine learning methods have also been widely applied. Linear regressions, including stepwise forward regressions, were applied in 8 out of 36 studies [11,13,15,24,29,37,38,41], including 3 out of 36 studies comparing their performance to estimations from neural networks [25,30,42]. Six out of 36 studies have applied other classics such as random forests, ensemble trees, and k-nearest neighbors [13,14,18,19,20,42]. Moreover, a few studies used alternative approaches, such as Oliveira et al. [26], who implemented a PCA combined with the conditional likelihood of the Fourier coefficients extracted from the sounds of footsteps. Weidersager et al. [39] applied ANOVA decomposition on pressure insole data, extracting multi-dimensional parameters that could reconstruct the vertical ground reaction force. Thirteen studies have evaluated more than one algorithm [12,13,14,18,19,20,22,25,30,34,35,42,44], with all studies except one [20] evaluating different neural network algorithms.

### 3.5. Variables Estimated from Machine Learning Algorithms

The vast majority of studies (25 out of 36 studies) applied machine learning methods to estimate vertical, horizontal, and lateral GRF [12,23,25,28,33,35,40,42,45,46] or only vertical GRF [11,17,19,20,22,26,27,31,32,34,36,38,39,43,44]. The other studies set machine learning algorithms to estimate GRF-based variables, such as impact peak, active peak, impulses, loading rates, and gait events to determine stance times (Table 2). A small subset of studies focused on classification-style outcomes (e.g., impact-related pattern classification or condition discrimination) using machine learning/deep learning frameworks [14,15].

### 3.6. Cross-Validation and Evaluation

Validation strategies varied substantially, with leave-one-subject-out (LOSO) approaches being used in 15 out of 36 studies (Figure 4B, Table 2). Dataset splits were used in seven studies [20,23,28,30,39,41,46]. Usual data splits used 65–80% data for training and 20–35% for testing. However, Cordero-Sánchez et al. [46] trained the models on only 35% of the data, since they used a publicly available dataset. Conversely, Johnson et al. [23] assessed a large biomechanical database from which ~19,400 trials were selected for training their models, applying their model in IMU data from five participants to predict GRF and moments. Sharma et al. [19] performed cross-validation by training the model with data from one participant and applying the model to the same participant and another unseen participant. Girka et al. [14] performed a 10-fold cross-validation, not explicitly mentioning whether the folds contained full participants or random data. Four studies applied multiple validations, such as LOSO and data splits [12,18,27], LOSO and leave-one-trial-out [13], and LOSO and subject-specific models trained with 50% of the participant’s data [15]. Five studies did not clearly describe their validations [11,22,29,34,42].

### 3.7. Performance Metrics

Thirty-four studies (94% of the total) predicted GRF curves or GRF-related variables using regression algorithms, and a variety of performance metrics were used. The vast majority of studies calculated absolute or relative errors between the experimental and predicted variables (Figure 4C). Absolute error metrics were usually the mean absolute error (MAE), mean square error (MSE), median absolute error, or root-mean square error (RMSE), whereas relative error metrics were the mean absolute percentage error (MAPE), relative (rRMSE), or normalized RMSE (nRMSE) (Table 3). Coefficient of correlations were applied in 69% of the studies, usually as an additional performance metric along with absolute or relative errors. The only exception was White et al., [11], who only used r metrics to estimate the performance of their GRF predictions. There were 13 studies extracting absolute/relative errors as well as associations/correlations [13,16,20,25,26,30,35,36,37,38,40,41]. Other techniques used to demonstrate the performance of the GRF predictions were Bland–Altman plots [30,31,32,36,37,40,44], Sensitivity analysis [14,21] and statistical parametric mapping (SPM, [25,33]. Two out of the 36 included studies predicted GRF with the purpose of classifications. Girka et al. [14] estimated the appearance of impact peaks, while Liu et al. [18] predicted the runner’s performance as novice, recreational, or competitive. Both studies assessed their classification performance using accuracy and/or F-measures and did not report any metric stating the quality of the GRF predictions.

### 3.8. General Outcomes from GRF Predictions

**Figure 5:**
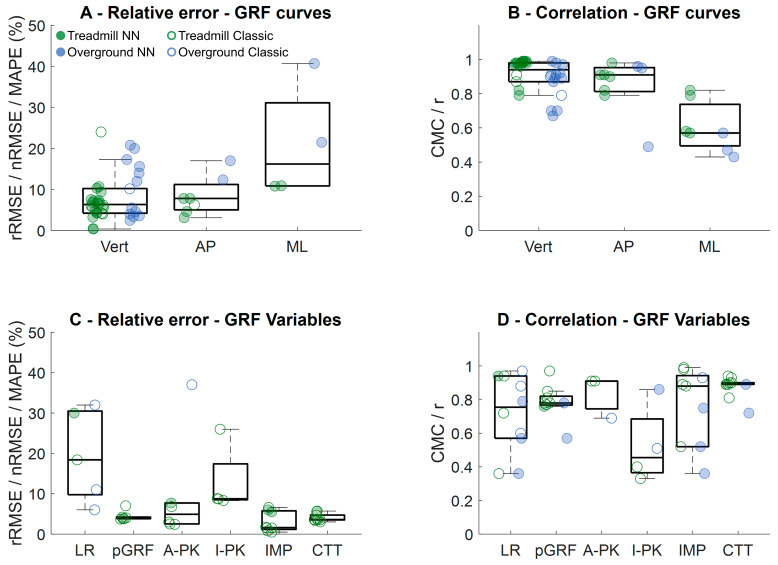
Distribution from studies evaluating relative errors (**A**) and their reported correlation/associations (**B**) from the prediction of vertical (Vert), anteroposterior (AP) and mediolateral (ML) ground reaction force (GRF) curves. In addition, distribution of studies evaluating GRF-based relative errors (**C**) and their reported coefficients of multiple correlation (CMC) or Pearson’s correlation coefficient (r) (**D**). Green circles represent treadmill studies; blue circles represent overground studies. Filled circles represent neural-network-based studies (NN), open circles represent classic machine learning algorithms (Classic). LR: loading rate; pGRF: peak vertical ground reaction force; A-PK: active peak; I-PK: impact peak; IMP: vertical impulse; CTT: foot contact time.

Studies predicting vertical GRF curves presented absolute errors varying from 0.02 to 0.70 BW (mean ± SD: 0.18 ± 0.12 BW). Predictions of anteroposterior and mediolateral GRF presented errors between 0.04–0.10 BW. Relative errors for GRF curve predictions were usually below 10% for both vertical and anteroposterior GRFs (Figure 5A), with correlation values > 0.8 (Figure 5B). However, the few studies reporting predictions of mediolateral GRF presented greater errors that reached up to 40% [45], as well as correlation values between 0.4–0.8. The majority of studies reporting performance of GRF curve predictions applied some type of neural network algorithm (Figure 5—filled circles). There were several studies reporting the prediction of GRF-based variables, most of them from treadmill-based studies (Figure 5—green circles). The results showed that loading rates presented high relative errors (median ~19%, Figure 5C), while peak vertical GRF, vertical impulses, active peaks, and foot contact time presented errors below 10% across studies. Following these results, correlation/association analyses demonstrated higher coefficients for peak GRF and foot contact time (Figure 5D). Despite the lower relative errors from vertical impulse, studies presented association values varying from 0.4–0.99.

## 4. Discussion

This scoping review summarizes current practices from 36 studies estimating ground reaction forces and GRF-derived variables during running using machine learning. Since Ancillao et al.’s [6] literature review on the topic, additional studies have provided further insights into predicting vertical GRF waveforms and several GRF-derived variables. However, there are limitations in terms of study quality, external validity, and reporting standards; all of which compromise between-study comparisons and may inflate expectations regarding real-world performance. In this discussion, we highlight some of the limitations and future perspectives for advancing the implementation of machine learning for GRF predictions.

### 4.1. Methodological Standard (Sex Imbalances, Sample Sizes, Running Speeds, Number of Steps)

We found that 59% of participants were male and 41% female, while ~52% of studies included ≤40% females. The imbalance is relevant as it limits the representativeness of females for data training. A systematic review by Xie et al. [47] demonstrated sex-specific changes in hip and knee angles, demonstrating that lower limb kinematics may differ between males and females. Such changes in running kinematics may be reflected in previously reported greater loading rates as well as propulsive and vertical forces during the late stance of running in females when compared to males [48,49]. Therefore, machine learning models trained using predominantly male data might not accurately represent female running dynamics. Our review identified a few studies with fewer than 20% females in their samples [12,19,26,43,46], and several studies not reporting sample distribution within both testing and training datasets [14,18,23,28,33,34,35]. Researchers accessing results from these studies should exercise caution when generalizing their findings to female runners. As we advance, reporting sex composition should be treated as essential, possibly with a balanced distribution between males/females. Moreover, case-by-case evaluation of subgroup performance (or inclusion of sex and anthropometric covariates) may be relevant for clearly establishing the validity/accuracy of machine learning models that can be used by both males and females.

Studies developing and/or validating machine learning models require sufficient amounts of data, but there are no clear standards regarding the distribution of within- and between-subject proportions. Oliveira and Pirscoveanu [50] reported a median of 21 runners across 51 studies on running biomechanics (mean: 31 runners). Van Hooren et al. [51] systematically reviewed the relation between running biomechanics and running economy, and the 40 studies included in the analysis presented a median of 17 participants (mean ± SD: 27 ± 19). Both studies demonstrate that mean values overestimate the general trend, since most of the studies involve 15 participants or fewer. Our review revealed a median of 18 participants per study and substantial variability (mean 33 ± 34), being in line with these reviews assessing large amounts of studies. However, a few studies presented 10 or fewer participants [11,19,34,36,38], including Johson et al. [23], who created a model with a large database, but tested it on only five participants. Moreover, some of these studies did not report the amount of data and/or steps being used in their models [11,34,36], making it difficult to replicate methods and assess the validity of the implementation. As described in the results, multiple studies do not clearly state the amount of data used for their model training and/or testing, and such omission compromises advances in the field. There is no clear guideline for sample size estimation considering GRF prediction, but based on the evidence from this review, studies assessing 18 participants—preferably with balanced sex distribution—will be on par with the median sample size from the field, and an increase to 33 participants can place the study on par with the mean sample size investigated in these previous studies.

GRF predictions during running have also accounted for different speeds/conditions. The majority of studies evaluate multiple running speeds, or in some cases incline/decline running [20,25]. Diversity in running speed is highly relevant for GRF prediction, since the curve pattern is influenced by running speed [52,53,54]. In this regard, literature has been progressing, since models are being generated to account for multiple speeds, despite some studies accessing a single speed in their experiments [11,14,26,39,46]. It is desirable that machine learning models can be transferable across different running speeds, and our review demonstrated that most of the studies assess running speeds between 2 m/s and 4 m/s, which may already include the preferred running speeds for most recreational runners. Studies included in this review evaluated the preferred running speed, which varied between 2.6 m/s to 3.7 m/s [14,26,29,38,40,45]. This matters because several modelling strategies implicitly assume adequate representation of each speed (and potentially each strike pattern) to prevent the model from learning speed-specific biases. In practice, sparse sampling per condition can lead to unstable performance estimates and can magnify the apparent success of approaches tested on narrow speed ranges.

### 4.2. Treadmill Versus Overground Running

A highly relevant result from our review was that the majority of studies (~58%) were conducted using treadmill running. These treadmill studies included more participants (median: 28) compared to overground running (median: 14), and the analyses were performed from more steps in treadmill studies (median: 65 steps/condition/participant) compared to overground running (median: 32 steps/condition/participant). The general outcome from these results indicates that studies developing/training machine learning models using treadmill running may present higher quality, as they have more participants and more data from each participant. It is widely accepted that machine learning models can only be generalizable by the inclusion of a considerable number of participants, each providing a fraction of the entire dataset [7,55]. Treadmill-based studies are more convenient to perform, facilitating continuous GRF acquisition, as well as a standardized way to manipulate running speed and or incline/decline modes. A few overground studies in our review recorded data from a high number of participants (ref. [14]: 135 participants, ref. [21]: 93 participants), but their protocols acquired fewer than 12 steps/participant. Other studies did the opposite, by extracting >90,000 steps for their analysis from fewer than 15 runners [31]. In contrast, there are treadmill studies acquiring a large number of participants and steps/participant [15,18]. Ideally, studies focusing on overground running need to increase both sample sizes and the number of acquired steps from each participant for a fair comparison to treadmill-based studies. Our results showed that these studies used a median of 65 steps/participant/condition for treadmill running and 32 steps/participant/condition for overground running, whereas the mean was between 86 to 139 steps/participant/condition, respectively. Therefore, studies focusing on GRF prediction during running may be on par with the recent literature by assessing ~65 steps/participant/condition (e.g., running speed) regardless of whether the study is on a treadmill or overground running.

Treadmill studies relied on instrumented devices to record ground truth GRFs. Although most overground studies used gold-standard force plates (11 out of 15; 73%), a non-trivial subset relied on pressure insoles [19,31,32,34]. Despite the clear advantage of acquiring estimated GRF at the highest ecological validity, insole data may present inaccuracies when compared to force plate measurements [56]. Moreover, insole recordings present limited sampling rates, sensor drift, calibration variability, and limited accuracy for non-vertical components [5]. Considering the difficulties in the acquisition of substantial amounts of steps from force plates in overground, and the technical limitations of pressure insoles, instrumented treadmills are currently the best option for high-quality research for predictions of GRF during running.

Currently, the real-world applicability of the proposed models to predict GRF data has not been attested. Firstly, the majority of predictions have been done using treadmill-based biomechanical motion patterns, as running data can be conveniently acquired from instrumented treadmills to generate such models. However, these models have not been tested using data from overground running and their transferability to real world condition cannot be assessed. Such transferability can be challenging, as outdoor running biomechanics can be influenced by wind resistance, surface/shoe compliance, changes in running speed and/or pace due to natural traffic conditions on the running path, and other factors. Secondly, only a few studies used plantar pressure insoles that truly allow running closest to natural conditions [19,31,32]. Despite not being the gold-standard technique for force measurements, plantar pressure insoles may be the best option to assess GRF during running in outdoors/real-world conditions. Improvements in pressure insole sensor quality and durability may elevate their usage to acquire GRF during outdoor running, potentially allowing studies with real-world applicability. Therefore, a major advance in this area may involve further development of insole technology, as well as the development of versatile models that can predict GRF from both treadmill and overground running.

### 4.3. Algorithmic Trends

Our review revealed that recent studies predicting GRF using machine learning are focusing on deep learning techniques (especially LSTM and Bi-LSTM) being applied to more convenient technologies—IMU sensors. Two studies included in our review [16,45] tested different numbers of IMUs for their predictions, ranging from one to seven sensors. Neither study found improvements in prediction accuracy when comparing a single shank-mounted IMU to predictions including more IMUs on trunk and other body segments. The trend of using deep-learning techniques is expected, as recurrent models are well-suited to underpin temporal dependencies within stance and can learn phase-related representations without extensive hand-crafted feature extraction. CNNs, MLPs, and FFNNs remain common, typically either as direct regressors or as components of hybrid pipelines [12,22,23,42,43]. However, it is noteworthy that classical regression approaches are still being applied and benchmarked against neural models, showing decent performance [11,13,15,16,29,38,41]. One advantage of linear models is that they have stronger interpretability and reduced risks of overfitting [57,58], which is particularly relevant given the variability in cohort size and reporting quality across the accessed literature. Taken together, the algorithmic landscape suggests that the field is not converging on a single “best” model class. Rather, the choice should be driven by the prediction target (waveform vs. scalar), the structure of the input (raw time-series vs. engineered features), and dataset size.

### 4.4. Validation and Generalization

One positive methodological trend in the literature is the emphasis on subject-independent evaluation, such as leave-one-subject-out (LOSO), highlighting that generalization across individuals is the preferred method for model validation. Dataset splits were also used in seven studies, typically with 65–80% training and 20–35% testing, and some studies applied multiple validation strategies to strengthen the findings. However, five studies did not clearly describe validation procedures [11,12,29,34,42], compromising the replication and interpretability of their results. The literature search demonstrates that highly individualistic models may not be relevant for future applications, despite studies creating subject-dependent models presenting greater accuracy when compared to subject-independent models [15,42]. It is noteworthy that LOSO validations performed within a single dataset should not be interpreted as equivalent to external validation, as training and test data often still share the same instrumentation, experimental protocol, pre-processing pipeline, and environmental constraints. As a result, reported accuracy may overestimate real-world performance.

LOSO approaches may be prone to overfitting when models are trained from relatively small and homogeneous cohorts with large numbers of steps per participant. Datasets with limited sample sizes but a high number of steps per runner will present low diversity, as consecutive steps from the same runner are highly correlated. In addition, treadmill running may further reduce intra-subject variability due to the highly controlled conditions when compared to overground running. Therefore, model performance may partly reflect sensitivity to study-specific patterns, especially running speed, sensor placement consistency, or laboratory-controlled conditions, rather than true robustness to unseen runners and environments. Under these conditions, especially when using deep neural networks, excellent predictive performance may partly reflect memorization of dataset-specific structure rather than learning of generalizable biomechanical relationships from truly unseen data. Future studies should focus on maximizing sample sizes to increase inter-subject variability and subsequent model generalization. Moreover, researchers must explicitly mention their sample sizes, the number of steps evaluated in the different conditions, and what type of validation strategy is being used. Furthermore, we found studies that did not report their validation procedure, and future studies must report their validation procedure (e.g., LOSO, data splits) to improve the quality of the experimental procedures and replicability.

### 4.5. Performance Results

We found that most studies report absolute and/or relative errors (e.g., RMSE/MAE; MAPE/rRMSE/nRMSE) and correlations (used in 69% of studies). However, at least one study relied on correlation metrics alone to report model performance [11]. A strong limitation of correlations/associations is that they do not account for data bias, which is highly undesirable when predicting GRF curves and/or variables. Absolute and relative errors are generally more interpretable for applied questions, while correlations are best treated as complementary indicators of waveform similarity or rank preservation [13,16,26,33,40]. Further analyses across participants, including bias (e.g., Bland–Altman plots) and sensitivity may help strengthen the findings. Encouragingly, several studies already include Bland–Altman plots [30,31,32,36,37,40,44] and sensitivity analyses [13,21,46] as complementary results. Moreover, deeper evaluation of curve patterns has been performed using SPM on a few studies [25,33,46], showing interesting potential for detailed comparisons when the predicted curves present high quality. Despite such exploratory statistical analyses, there is a concerning heterogeneity in the reported metrics across studies. For instance, 19 studies reported RMSE or MAE to describe absolute errors when predicting GRF curves, while 16 studies reported relative errors (relative RMSE and MAPE) from their GRF curve predictions. It is encouraging that we identified 13 studies that reported absolute errors, relative errors, and correlations/associations in their results [13,16,24,25,26,30,35,36,37,38,40,41,44], demonstrating that some studies use multiple data analyses to evaluate their models. Moreover, 27 studies reported direct GRF curves, while 17 studies reported GRF-derived metrics such as loading rates, peak forces, or impulses. Such heterogeneity limited our power to explore the data, for instance by exploring the association between the number of IMU sensors and the GRF prediction quality. Interestingly, we identified 10 studies that evaluated both GRF curves and GRF-based variables [11,17,24,26,29,31,32,37,38,40], but such detailed data analysis was achieved only by a fraction of the studies assessed in the review. Therefore, future studies should focus on providing metrics describing both absolute and relative errors from GRF curves, with correlation analyses being supplementary due to their lack of sensitivity to data scaling. Researchers interested in GRF-based metrics should also report the prediction quality from both absolute and relative errors. Further analysis using bias, sensitivity, or GRF curve region-specific comparisons may be applied when the research context requires such details.

### 4.6. Overview of Outcome Measures

The general outcomes indicate vertical GRF curve predictions present absolute errors ranging from 0.02 to 0.70 BW (mean 0.18 ± 0.12 BW), relative errors were usually below 10% and correlations were typically >0.8. The overall trend suggests that vertical GRF prediction quality is moving toward supporting monitoring applications, such as treadmill training monitoring and/or lab-based biomechanics augmentation. In contrast, estimation of anteroposterior and mediolateral GRF remains challenging, showing higher errors (up to ~40%) and lower correlations (0.4–0.8), especially in the mediolateral direction [12,23,25,33,42,43,45,46]. The issue may be more related to measurement factors than algorithm quality, since mediolateral forces present a lower magnitude and are more sensitive to subtle changes in foot placement. Moreover, current setups using a single foot IMU may not be able to capture the complexity of 3D foot dynamics that involves multiple foot segments, compromising mediolateral GRF prediction quality. The results from this review demonstrate that further studies implementing better acquisition methods to capture intricacies from mediolateral GRF are necessary to improve prediction quality.

A consistent result across studies targeting GRF-derived variables is that performance depends strongly on the variable’s nature, as peak-related measures (peak vertical GRF; impact/active peaks), impulses, and foot contact time generally show relative errors below 10% across studies and tend to exhibit higher associations [11,16,21,26,38,40,41]. These variables are either dominated by lower-frequency waveform structure (peaks/impulses) or are tied to event timing that can be reliably inferred from kinematic/IMU signatures in many protocols. Unsurprisingly, loading rates show markedly higher errors (median ~19%) that may be considered as a methodological and biomechanical limitation rather than a failure of any specific algorithm. Small timing shifts have limited influence on peak GRF or impulse and can produce large relative errors in loading rate calculations [14,21,25,33]. Moreover, the sampling and filtering pipelines used for both force and sensor signals can influence loading rates. Especially the relatively lower sampling rates from IMUs (100–240 Hz) may be a crucial limitation when estimating loading rates within 20–50 ms windows. Further studies focusing on predictions of loading rates should explore different machine learning model structures, expand input feature selection and explore different venues for biomechanical data pre-processing methods towards improving prediction accuracies.

### 4.7. Recommendations for Future Work

As a scoping review, the present synthesis emphasizes trends and gaps rather than pooling outcomes under a single benchmark. Nonetheless, the recurring patterns identified across studies allow us to highlight priorities for future research aimed at improving study quality and comparability:

1. In addition to reporting sample sizes, studies should report the male/female distribution and training status of the participants. While an objective recommendation for sample size is not suitable for this scoping review, our results revealed that the mean sample size was 33 participants for studies investigating treadmill running. Since model generalization requires data variability from different individuals, researchers should plan research protocols to include appropriate sample sizes.
2. Experimental protocols should prioritize assessments of multiple running speeds to broaden algorithms exposure to different GRF patterns. This is more conveniently achievable in treadmill settings, but efforts must be made to increase the standards in overground studies. Experimental designs should be scaled to warrant appropriated number of steps from whichever number of speeds evaluated. Our results revealed that the mean number of steps per participant per condition was ~65 steps, serving as a possible baseline for the planning of data collections.
3. There has been no tangible real-world applicability of current models dedicated to predicting GRF during running. Researchers may focus on creating large-scale models that are trained from treadmill running and tested on overground running. Successful transferability of treadmill-based models towards real-world running biomechanics will be a breakthrough in the field. Moreover, more studies evaluating GRF assessment using plantar pressure insoles may help advancing towards real-world applications, since such instrumentation allows recordings in natural running conditions.
4. The use of deep learning for GRF prediction has increased, following general trends in most of the engineering/medical fields. However, classical machine learning algorithms usually present comparable performance and may be advantageous for limited sample sizes and reduced risks of overfitting. Researchers should consider whether sophisticated deep neural networks are necessary for their applications, since such models take longer to train and require deeper methodological skills to establish correct hyperparameters.
5. The prediction of vertical GRF presents acceptable errors, whereas anteroposterior and especially mediolateral GRFs still need further development. Peak forces and impulses can be accurately predicted, but variables that depend on short data sectors, such as loading rates, are still not adequately predicted. Researchers interested in extracting GRF-based variables should consider exploring different prediction models with different feature selections to improve prediction accuracies.

## 5. Conclusions

With the increased demand for data-driven training/rehabilitation programs, accurate estimations of ground reaction forces during running have strong potential to help practitioners improve their performance and/or detect abnormal patterns that may lead to injuries. The findings gathered in this literature review demonstrate that predictions of vertical GRF are increasingly reliable, but more studies are necessary for progress in the prediction of anteroposterior and mediolateral GRF. Studies have predominantly predicted GRF during treadmill settings on samples of predominantly male runners, limiting possible generalization towards real-world running conditions. Here, major advances are required, since no study has attempted to cross-validate models on both treadmill and overground running. Finally, despite the increased use of deep learning/neural network algorithms, classical regression methods may still be relevant depending on the application and/or limitations on the study datasets.

## Figures and Tables

**Table 1:** Overview of ground truth, used sensors, sample size, age, training status, running speed, and number of steps used in the papers found.

Study	Ground Truth	Prediction Sensors	# Sensors	Sample Size	% Females	Age (Average)	Training Status	Running Speed	Steps/Runner/ Speed
**White (2019) [11]**	Instr. treadmill	Markers	16 MRK	10	60%	15.5	4	3.10	NR
**Komaris (2019) [12]**	Instr. treadmill	Markers	8 MRK	28	4%	38.4	4	2.5–4.5	NR
**Derie (2020) [13]**	Force plate	IMUs	2 IMU	93	41%	35.2	2	2.5–5.1	14
**Girka (2020) [14]**	Force plate	Markers	NR	135	NR	NR	4	3.70	9
**Seeley (2020) [15]**	Instr. treadmill	Instr shoe + IMU	4 PS, 1 IMU	31	45%	23	3	2.7–3.5	309
**Tan (2020) [16]**	Instr. treadmill	IMUs	1–5 IMU	15	47%	23.9	NR	2.4–2.8	500
**Pogson (2020) [17]**	Force plate	IMUs	1 IMU	15	33%	23	3	2.0–8.0	6
**Liu (2020) [18]**	Instr. treadmill	IMUs	2 IMUs	30	47%	31.3	1	1.9–4.4	65
**Sharma (2021) [19]**	Insole	Other (INS/GPS)	1	2	0%	26.5	NR	2.0–6.0	32
**Alcantara (2021) [20]**	Instr. treadmill	IMUs	1 IMU	50	76%	20	1	3.8–4.9	8
**Robberechts (2021) [21]**	Force plate	IMUs	2 IMUs	93	41%	35.2	3	2.5–5.1	3
**Tedesco (2021) [22]**	Insole	IMUs	2 IMUs	14	29%	29	NR	2.2–3.3	65
**Johnson (2021) [23]**	Force plate	IMUs	3 IMUs	5	20%	NR	4	2.5–6.0	NR
**Alcantara (2022) [24]**	Instr. treadmill	IMUs	3 IMUs	19	47%	29	NR	2.5–4.1	65
**Honert (2022) [25]**	Instr. treadmill	Other (insole)	99 PS	18	50%	28	2	2.6–3.8	346
**Oliveira (2022) [26]**	Force plate	Other (MIC)	4 MICs	44	18%	26	2	2.75	40
**Bach (2022) [27]**	Instr. treadmill	IMUs	2 IMUs	21	38%	20.8	NR	2.2	72
**Candela-Leal (2022) [28]**	Instr. treadmill	Markers	32 MRK	28	NR	NR	3	2.5–4.5	NR
**Martinez (2022) [29]**	Instr. treadmill	Other (2D video)	1 CAM	30	57%	31.8	3	3.1	NR
**Patoz (2023) [30]**	Instr. treadmill	IMUs	1 IMU	100	27%	29.5	2	2.5–3.6	3
**Donahue (2023) [31]**	Insole, GPS	IMUs	3 IMU	13	46%	23.2	NR	2.2–5.2	541
**Donahue (2023) [32]**	Insole, GPS	IMUs	3 IMU	15	40%	23.6	NR	2.2–5.3	NR
**Mundt (2023) [33]**	Force plate, Camera	Other (2D video)	1 CAM	14	100%	23	2	4.5–5.5	NR
**Zhu (2023) [34]**	Insole /MOCAP	IMUs	5 IMUs	10	NR	NR	NR	NR	NR
**Scheltinga (2023) [35]**	Instr. treadmill	IMUs	3 IMUs	12	NR	NR	3	2.8–3.8	13
**Wang (2023) [36]**	Instr. treadmill	Markers	NR	9	67%	21	3	3.3–4.4	5
**Veras (2023) [37]**	Instr. treadmill	IMUs	3 IMUs	131	40%	32	NR	1.9–3.9	NR
**Provot (2023) [38]**	Force plate	IMUs	2 IMUs	9	56%	23	2	2.7–3.4	110
**Weidensager (2024) [39]**	Instr. treadmill	Other (insoles)	8 PS	18	28%	29.5	NR	2.5	37
**Carter (2024) [40]**	Instr. treadmill	Other (insole + IMU)	16 PS	50	50%	33	2	3.0–3.3	NR
**Bogaert (2024) [41]**	Instr. treadmill	IMUs	1 IMU	43	28%	24	NR	2.2–3.3	360
**Song (2025) [42]**	Instr. treadmill	IMUs	3 IMUs	44	57%	NR	4	3.8–4.9	NR
**Chen (2025) [43]**	Force plate	IMUs	NR	12	0%	22	NR	2.2–4.4	9
**Li (2025) [44]**	Instr. treadmill	IMUs	1 IMU	15	47%	21	4	3.3–4.4	100
**Yilmazgün (2025) [45]**	Force plate	IMUs	1–7 IMUs	20	50%	24	NR	2.5–2.7	67
**Cordero-Sánches (2025) [46]**	Force plate	IMUs + Markers	NR	11	0%	30	NR	3.3	110

Abbreviations: Instr: instrumented; Insole: Plantar pressure shoe insole; MIC: professional microphones; IMU: inertial measurement unit; MOCAP: motion capture; MRK: retro-reflexive markers; PS: pressure sensors; CAM: 2D video camera; NR: not reported information. Training status tiers: (1) sedentary/untrained/novice; (2) recreational runner (runner practitioner without specific training regimen); (3) experienced (with many years of practice and specific training regimen); (4) high-level/professional runner. Bold text is used to distinguish the columns and rows.

**Table 2:** Machine learning approaches for GRF/kinetic prediction across studies.

Study	Algorithm Name	Data Split/Validation	Prediction Target (GRF/Kinetics)
**White (2019) [11]**	Stepwise forward LR	unclear	Peak vertGRF; average vertical loading rate
**Komaris (2019) [12]**	FFNN/MLP	LOSO within training set, split 60/20/20 (train/valid/test);	Vertical, AP and ML GRF curves
**Derie (2020) [13]**	LR (LASSO), Elastic Net, XGBoost	LOSO (subject-independent) and leave-one-trial-out within subject (subject-dependent)	Loading rate
**Girka (2020) [14]**	Convolutional NN, Random Forest	10-fold cross-validation	Impact peak presence/absence in vertGRF
**Seeley (2020) [15]**	PCR; LR	General model: LOSO; Subject-specific models: within-subject training split (~50% training)	vertGRF curve, Active peak, impact peak, loading rate, impulse, contact time
**Tan (2020) [16]**	Convolutional NN	LOSO	Loading rate
**Pogson (2020) [17]**	PCA + MLP	Subject-separated train/test (7 train/8 test) repeated 10× (random splits)	vertGRF curve, impact peak, loading rate, impulse
**Liu (2020) [18]**	CNN, MLP, GBDT	Split 80/20 (train/test); Leave-one-out	Loading rate; peak braking force; peak propulsion force
**Sharma (2021) [19]**	LSTM, bagged trees, KNN	Train on one subject; test on the same subject and on a second unseen subject	Vert GRF curve, peak vert GRF, impulse
**Alcantara (2021) [20]**	QRF and LR	Split 76/24 (train/test)	Peak vertGRF; vertical impulse; contact time
**Robberechts (2021) [21]**	Bi-LSTM	LOSO	Initial contact, toe-off, stance time
**Tedesco (2021) [22]**	Feed-forward NN, MLP	Unclear	vertGRF curve, peak GRF
**Johnson (2021) [23]**	Convolutional NN	Split ~95/5 (training/test)	Vertical, AP and ML GRF curves and moments
**Alcantara (2022) [24]**	Bi-LSTM, MLP	LOSO	vertGRF curve, active peak, impulse, loading rate, contact time
**Honert (2022) [25]**	Bi-LSTM, LR	LOSO	Vertical and AP GRF curves
**Oliveira (2022) [26]**	PCA + Fourier transformation	LOSO	vertGRF curve
**Bach (2022) [27]**	Echo State Network (ESN)	LOSO, Split 50/25/25 (train/valid/test)	vertGRF curve
**Candela-Leal (2022) [28]**	LSTM	Split 70/10/20 (train/valid/test)	Vertical, AP and ML GRF curves
**Martinez (2022) [29]**	Stepwise-forward LR	Unclear	Peak vertGRF, loading rate, peak braking force
**Patoz (2023) [30]**	LIN-REG, SVR, 2-layer NN	Split 80/20 (train/test)	Peak vertGRF
**Donahue (2023) [31]**	Bi-LSTM	LOSO	vertGRF curve, Peak GRF, impulse, loading rate, contact time
**Donahue (2023) [32]**	LSTM	LOSO	vertGRF curve, Peak GRF, impulse, loading rate, contact time
**Mundt (2023) [33]**	Bi-LSTM	LOSO	3D GRF curves
**Zhu (2023) [34]**	Transformer encoder, RNN, LSTM	Unclear	vertGRF curve
**Scheltinga (2023) [35]**	FFNN, MLP, Physics-based model	LOSO	Vertical, AP and ML GRF curves
**Wang (2023) [36]**	WNN	LOSO	vertGRF curve, impact peak, active peak, peak timing
**Veras (2023) [37]**	Linear mixed-model regression	LOSO	Peak GRF and peak loading rate
**Provot (2023) [38]**	Stepwise multiple LR	Trained/validated on 1 subject; tested on 8 new participants	vertGRF curve, passive/active peaks, loading rate
**Weidensager (2024) [39]**	ANOVA decomposition	Split 80/20 (train/valid)	vertGRF curve
**Carter (2024) [40]**	Bi-LSTM	LOSO	Vertical and AP GRF curves, peak GRF, impulse, braking and propulsive forces
**Bogaert (2024) [41]**	LR (LASSO)	Split 80/20 (train/valid)	Active peak, impact peak, impulse, contact time
**Song (2025) [42]**	SER, KNN, LSTM, Bi-LSTM	Unclear	Vertical, AP and ML GRF curves
**Chen (2025) [43]**	LSTM, extended LSTM	LOSO	vertGRF curves
**Li (2025) [44]**	Wavelet NN, feed-forward NN, MLP	LOSO	vertGRF curves, peak GRF
**Yilmazgün (2025) [45]**	Convolutional NN	LOSO	Vertical, AP and ML GRF curves
**Cordero-Sánches (2025) [46]**	LSTM	Split 35/65 (train/test) *	Vertical, AP and ML GRF curves

Abbreviations: LR: linear regression; FFNN: feed-forward neural network; MLP: multi-layer perceptron; XGBoost: extreme gradient boosting; convolutional NN: convolutional neural network; PCR: principal component regression; PCA: principal component analysis; CNN: convolutional neural network; GBDT: Gradient Boosted Decision Trees; LSTM: long short-term memory network; KNN: K-nearest neighbors; QRF: quantile random forest; Bi-LSTM: bidirectional long short-term memory network; feed-forward NN: feed-forward neural network; ESN: Echo State Network; LIN-REG: linear regression; SVR: support vector regression; RNN: recurrent neural network; WNN: wavelet neural network; ANOVA: analysis of variance; SER: standard error of regression; LASSO: least absolute shrinkage and selection operator; LOSO: leave-one-subject-out cross-validation. * Indicates that the study used the training data from one dataset and the testing data from another dataset. Bold text is used to distinguish the columns and rows.

**Table 3:** Overview of prediction performance metrics: absolute errors (RMSE, MAE), relative errors (rRMSE, nRMSE, MAPE), and correlation coefficients (r, r^2^).

Study	Absolute Errors (RMSE/MAE)	Relative Errors (rRMSE, nRMSE, MAPE)	Correlation (r, r^2^)
**White (2019) [11]**	RMSE vert GRF curve: 0.3 BW		r^2^ Contact time: 0.79
			r^2^ Stance force: 0.37
			r^2^ Impulse: 0.27
			r^2^ Peak force 0.61
			r^2^ Loading rate: 0.13
**Komaris (2019) [12]**	RMSE vert GRF: 0.13 BW		
	RMSE AP GRF: 0.041 BW		
	RMSE ML GRF: 0.042 BW		
**Derie (2020) [13]**	MAE SUB-IND VILR: 5.4 BW·s	MAPE SUB-IND VILR: 6%	r^2^ SUB-IND VILR: 0.94
	MAE SUB-DEP VILR: 12.4 BW·s	MAPE SUB-DEP VILR: 11%	r^2^ SUB-DEP VILR: 0.77
**Girka (2020) [14]**	Classification task		
**Seeley (2020) [15]**	RMSE SUB-IND Active peak: 129.8 N	rRMSE SUB-IND Active peak: 6.8%	
	RMSE SUB-IND Impulse: 17.7 N·s	rRMSE SUB-IND Impulse: 6.6%	
	RMSE SUB-IND contact time: 0.01 s	rRMSE SUB-IND contact time:5.7%	
	RMSE SUB-IND impact peak: 317 N	rRMSE SUB-IND impact peak: 26%	
	RMSE SUB-IND impact rate: 10.5 kN/s	rRMSE SUB-IND impact rate: 29%	
	RMSE SUB-DEP Active peak: 59 N	rRMSE SUB-DEP Active peak: 3%	
	RMSE SUB-DEP Impulse: 5.4 N·s	rRMSE SUB-DEP Impulse: 1.7%	
	RMSE SUB-DEP contact time: 0.01 s	rRMSE SUB-DEP contact time: 3.6%	
	RMSE SUB-DEP impact peak: 122.7 N	rRMSE SUB-DEP impact peak: 8.8%	
	RMSE SUB-DEP impact rate: 8.5 kN/s	rRMSE SUB-DEP impact rate: 25.5%	
**Tan (2020) [16]**	Single IMU MAE VALR: 13.8 BW/s	Single IMU nRMSE VALR: 9.7%	Single IMU r VALR: 0.94
**Pogson (2020) [17]**	RMSE GRF curve: ~0.20–0.25 kN		r^2^ impact peak: 0.74
			r^2^ loading rate: 0.63
			r^2^ Impulse: 0.13
**Liu (2020) [18]**	Classification study		
**Sharma (2021) [19]**		NRMSE Same subject: 1–7% across variables	
		nRMSE across subjects: 1–23% across variables	
**Alcantara (2021) [20]**	QRF RMSE peak vertical GRF: 0.15 BW,	QRF MAPE peak vertical GRF: 4.2%	QRF r peak vertical GRF: 0.81
	QRF RMSE impulse: 0.004 BW·s,	QRF MAPE impulse: 0.8%	QRF r impulse: 0.98
	QRF RMSE contact time: 0.011 s	QRF MAPE contact time: 4.7%	QRF r contact time: 0.81
	LR RMSE peak vertical GRF: 0.14 BW,	LR MAPE peak vertical GRF: 4.0%	LR r peak v vertical GRF: 0.85
	LR RMSE impulse:0.002 BW·s,	LR MAPE impulse: 0.5%,	LR r impulse:0.99
	LR RMSE contact time: 0.008 s	LR MAPE contact time: 3.5%	LR r contact time: 0.90
**Robberechts (2021) [21]**	RNN MAE stance time: 6.5 ms		
	Perceptron MAE stance time: 10 ms		
	Baseline MAE stance time: 11.3 ms		
**Tedesco (2021) [22]**	RMSE GRF curve 8 km/h: 0.13 BW		
	RMSE GRF curve 10 km/h: 0.13 BW		
	RMSE GRF curve 12 km/h: 0.17 BW		
**Johnson (2021) [23]**		rRMSE vert GRF: 14%	Mean r CaffeNet: 0.89
		rRMSE AP GRF: 17%	Mean r ResNet: 50: 0.87
		rRMSE ML GRF: 21.5%	
Alcantara (2022) [24] * results only from LSTM without foot strike	RMSE GRF curve: 0.17 BW	rRMSE GRF curve: 6.7%	
		MAPE step frequency: 0.1%	
		MAPE contact time: 5.6%	
		MAPE Impulse: 6%	
		MAPE Active peak: 7.7%	
		MAPE loading rate: 30%	
**Honert (2022) [25]**	BiLSTM RMSE vert GRF curve: ~0.12–0.18 BW	BiLSTM RMSE vert GRF curve: ~4–5.5%	Vert GRF: 0.98–0.99+
	Linear RMSE vert GRF curve: ~0.22–0.29 BW	Linear RMSE vert GRF curve: ~5.6–6.7%	AP GRF: 0.85–0.95
	BiLSTM RMSE AP GRF curve: ~0.03–0.07 BW	BiLSTM RMSE AP GRF curve: ~4.2–5.0%	
	Linear RMSE AP GRF curve: ~0.07–0.13 BW	Linear RMSE AP GRF curve: ~5.8–6.7%	
**Oliveira (2022) [26]**	Vert GF RMSE curve: 0.25 BW	Vert GF rRMSE curve: ~5.7–14.6%	Mean r vert GRF curve r: 0.9
	RMSE impact peak: 0.22 BW		r impact peak: 0.51
	RMSE active peak: 0.12 BW		r active peak: 0.69
	RMSE loading rate: ~14.46 BW/s		r loading rate: ~0.60
	RMSE impulse: 5.2 BW·s		r impulse: 0.93
**Bach (2022) [27]**		nRMSE vert GRF curve: 6.8%	r vert GRF curve: 0.96
**Candela-Leal (2022) [28]**			Mean r LSTM up-sampling GRF (x, y z): 0.82
			Mean r LSTM down-sampling GRF (x, y z): 0.79
**Martinez (2022) [29]**			r^2^ vert GRF curve: 0.75
			r^2^ loading rate: 0.52
			r^2^ peak braking force: 0.55
**Patoz (2023) [30]**	RMSE LR contact time: 11.9 ms	MAPE LR contact time: 3.6%	r LR contact time: 0.90
	RMSE LR peak GRF: 0.12 BW	MAPE LR peak GRF: 3.7%	r LR peak GRF: 0.78
	RMSE SVR contact time: 12.3 ms	MAPE SVR contact time: 3.6%	r SVR contact time: 0.89
	RMSE SVR peak GRF: 0.13 BW	MAPE SVR peak GRF: 3.8%	r SVR peak GRF: 0.77
	RMSE NN contact time: 12.3 ms	MAPE NN contact time: 3.7%	r NN contact time: 0.89
	RMSE NN peak GRF: 0.13 BW	MAPE NN peak GRF: 3.8%	r NN peak GRF: 0.76
**Donahue (2023) [31]**	RMSE vert GRF curve: ~0.23–0.64 BW		r^2^ contact time: ~0.52
	RMSE contact time: ~0.02–0.04 s		r^2^ peak GRF: ~0.33
	RMSE peak GRF: ~0.12–0.51 BW		r^2^ impulse: ~0.57
	RMSE impulse: 0.02–0.09 BW·s		r^2^ loading rate: ~0.16–0.49
	RMSE loading rate: 12–45 BW/s		
**Donahue (2023) [32]**	RMSE vert GRF curve: ~0.19–0.30 BW		r^2^ contact time: ~0.79
	RMSE contact time: ~0.02–0.09 s		r^2^ peak GRF: ~0.61
	RMSE peak GRF: ~0.16–0.26 BW		r^2^ impulse: ~0.27
	RMSE impulse: 0.03–0.10 BW·s		r^2^ loading rate: ~0.13
	RMSE loading rate: ~7.8–18 BW/s		
**Mundt (2023) [33]**		nRMSE AlphaPose GRF curve: ~0.36–0.42%	r AlphaPose GRF curve: ~0.63–0.76
		nRMSE BlazePose GRF curve: ~0.27–0.42%	r BlazePose GRF curve: ~0.65–0.69
		nRMSE OpenPose GRF curve: ~0.36–0.50%	r OpenPose GRF curve: ~0.60–0.80
**Zhu (2023) [34]**	MSE RNN vert GRF curve: 0.036	MAPE RNN vert GRF curve: 84.4%	
	MSE LSTM vert GRF curve: 0.029	MAPE LSTM vert GRF curve: 82.7%	
	MSE Transformer vert GRF curve: 0.021	MAPE Transformer vert GRF curve: 79.2%	
	MSE Transformer + GATE_MSE vert GRF curve: 0.022	MAPE Transformer + GATE_MSE vert GRF curve: 80.0%	
**Scheltinga (2023) [35]**	Hybrid RMSE vert GRF curve: 0.18 BW	Hybrid rRMSE vert GRF curve: 6.8%	Hybrid r vert GRF curve: 0.97
	Hybrid RMSE AP GRF curve: 0.07 BW	Hybrid rRMSE AP GRF curve: 7.8%	Hybrid r AP GRF curve: 0.91
	Hybrid RMSE ML GRF curve: 0.05 BW	Hybrid rRMSE ML GRF curve: 10.8%	Hybrid r ML GRF curve: 0.58
	Direct RMSE vert GRF curve:0.19 BW	Direct rRMSE vert GRF curve: 7.3%	Direct r vert GRF curve: 0.97
	Direct RMSE AP GRF curve: 0.08 BW	Direct rRMSE AP GRF curve: 7.8%	Direct r AP GRF curve: 0.91
	Direct RMSE ML GRF curve: 0.05 BW	Direct rRMSE ML GRF curve: 10.9%	Direct r ML GRF curve: 0.57
**Wang (2023) [36]**	RMSE vert GRF curve 12 km/h: 0.28 BW	nRMSE vert GRF curve 12 km/h: 9.4%	CMC vert GRF curve 12 km/h: 0.98
	RMSE vert GRF curve 14 km/h: 0.33 BW	nRMSE vert GRF curve 14 km/h: 10.3%	CMC vert GRF curve 14 km/h: 0.98
	TMSE vert GRF curve 16 km/h: 0.24 BW	nRMSE vert GRF curve 16 km/h: 10.7%	CMC vert GRF curve 16 km/h: 0.98
**Veras (2023) [37]**	RMSE peak GRF: ~130–137 N	MAPE peak GRF: ~6.7–7.3%	r^2^ peak GRF > 0.95
	RMSE peak loading rate: ~3971–4619 N	MAPE peak loading rate: ~17.7–19.1%	r^2^ peak loading rate: ~0.87–0.91
	MAE peak GRF: ~93–100 N		
	MAE peak loading rate: ~2882–3249 N		
**Provot (2023) [38]**	RMSE vert GRF curve: ~0.4–1.0 BW	DRD active peak: ~16–58%	r^2^ GRF curve: ~0.29–0.95
		DRD loading rate: ~15–49%	
**Weidensager (2024) [39]**		NRMSE vertical GRF curve 9 km/h: 0.24 (24%)	r vert GRF curve: 0.91
**Carter (2024) [40]**	RMSE vert GRF curve: 0.15 BW	rRMSE vert GRF curve: 3.2%	r vert GRF curve: 0.99
	RMSE AP GRF curve: 0.04 BW	rRMSE AP GRF curve: 3.1%	r PA GRF curve: 0.98
	MAE peak braking force: ~0.03 BW	%difference peak impulse error: 6.3%	
	MAE peak propulsion force: ~0.03 BW	%difference impulse: 5.5%	
**Bogaert (2024) [41]**	RMSE no speed active peak: 0.08 BW	MAPE no speed active peak: 2.4%	r^2^ no speed active peak: 0.83
	RMSE no speed impact peak: 0.19 BW	MAPE no speed impact peak: 8.3%	r^2^ no speed impact peak: 0.16
	RMSE no speed impulse: 0.007 BW·s	MAPE no speed impulse: 1.5%	r^2^ no speed impulse: 0.79
	RMSE no speed contact time: 0.01 s	MAPE no speed contact time: 3%	r^2^ no speed contact time: 0.89
	RMSE with speed active peak: 0.08 BW	MAPE with speed active peak: 2.5%	r^2^ with speed active peak:0.82
	RMSE with speed impact peak: 0.20 BW	MAPE with speed impact peak: 8.7%	r^2^ with speed impact peak:0.11
	RMSE with speed impulse: 0.007 BW·s	MAPE with speed impulse: 1.5%	r^2^ with speed impulse: 0.78
	RMSE with speed contact time: 0.01 s	MAPE with speed contact time: 3.3%	r^2^ with speed contact time:0.87
**Song (2025) [42]**	SER RMSE SUB-IND vert GRF curve: 0.19 BW	SER RMSE SUB-IND vert GRF curve: 6.5%	
	KNN RMSE SUB-IND vert GRF curve: 0.18 BW	KNN RMSE SUB-IND vert GRF curve: 6%	
	LSTM RMSE SUB-IND vert GRF curve: 0.12 BW	LSTM RMSE SUB-IND vert GRF curve:4.2%	
	SER RMSE SUB-DEP vert GRF curve: 0.13 BW	SER RMSE SUB-DEP vert GRF curve: 4.2%	
	KNN RMSE SUB-DEP vert GRF curve: 0.12 BW	KNN RMSE SUB-DEP vert GRF curve: 4%	
	LSTM RMSE SUB-DEP vert GRF curve: 0.13 BW	LSTM RMSE SUB-DEP vert GRF curve: 4.3%	
Chen (2025) [43] * results are range across all speeds	RMSE 3J/3P vert GRF curve: ~0.06–0.07 BW	MAPE 3J/3P vert GRF curve: ~2.2–2.7%	r^2^ 3J/3P vert GRF curve: ~0.83–0.88
	RMSE Ank/3P vert GRF curve: ~0.10–0.12 BW	MAPE Ank/3P vert GRF curve: ~4.4–4.8%	r^2^ Ank/3P vert GRF curve: ~0.79–0.81
	RMSE 3J/Sag vert GRF curve: ~0.08–0.10 BW	MAPE 3J/Sag vert GRF curve: ~3.2–3.9%	r^2^ 3J/Sag vert GRF curve: ~0.81–0.83
	RMSE 3J/Front vert GRF curve: ~0.07–0.09 BW	MAPE 3J/Front vert GRF curve: ~3.0–3.7%	r^2^ 3J/Front vert GRF curve: ~0.80–0.85
**Li (2025) [44]**	RMSE WNN model three axes: 0.15–0.21 BW	nRMSE WNN model three axes: 4.5–6.7%	r^2^ WNN model three-axis: ~0.93–0.98
	RMSE WNN model sagittal axis: 0.16–0.22 BW	nRMSE WNN model sagittal axis: 4.6–6.8%	r^2^ WNN model sagittal-axis: ~0.94–0.98
	RMSE FFNN model three axes: 0.19–0.29 BW	nRMSE FFNN model three axes: 5.9–9.2%	r^2^ FFNN model three-axis: ~0.96–0.99
	RMSE FFNN model sagittal axis: 0.0.19–0.28 BW	nRMSE FFNN model sagittal axis: 5.7–8.8%	r^2^ FFNN model sagittal axis: ~0.96–0.99
**Yilmazgün (2025) [45]**		RRMSE running vert GRF curve: ~4.5–6.5%	r running vert GRF curve: 0.99
		RRMSE running AP GRF curve: ~10.3–14.4%	r running AP GRF curve: 0.94–0.97
		RRMSE running ML GRF curve: ~32.1–49.3%	r running ML GRF curve: 0.46–0.68
**Cordero-Sánches (2025) [46]**	RMSE ANN vert GRF curve: 1.7–2.6 N/kg		r ANN vert GRF curve: 0.96–0.99
	RMSE Physics vert GRF curve: 1.8–3.0 N/kg		r Physics vert GRF curve: 0.96–0.97
	RMSE ANN AP GRF curve: 0.6–1.2 N/kg		r ANN AP GRF curve: 0.93–0.97
	RMSE Physics AP GRF curve: 0.6–2.4 N/kg		r Physics AP GRF curve: 0.32–0.65
	RMSE ANN ML GRF curve: 0.3–0.55 N/kg		r ANN ML GRF curve: 0.28–0.65
	RMSE Physics ML GRF curve: 0.4–1.10 N/kg		r Physics ML GRF curve: 0.34–0.52

Abbreviations: GRF: ground reaction force; vert GRF: vertical ground reaction force; RMSE: root mean square error; BW: body weight; r^2^: coefficient of determination; MAE: mean absolute error; VILR: vertical instantaneous loading rate; MAPE: mean absolute percentage error; SUB-IND: subject-independent; SUB-DEP: subject-dependent; rRMSE: relative root mean square error; kN/s: kilonewtons per second; AP GRF: anterior–posterior ground reaction force; ML GRF: medio–lateral ground reaction force; VALR: vertical average loading rate; nRMSE: normalized root mean square error; r: Pearson correlation coefficient; QRF: quantile random forest; BW·s: body weight–seconds; LR: linear regression; RNN: recurrent neural network; LSTM: long short-term memory network; BiLSTM: bidirectional long short-term memory; SVR: support vector regression; NN: neural network; MSE: mean squared error; DRD: dynamic range difference; AlphaPose: pose estimation model; BlazePose: pose estimation model; OpenPose: pose estimation model; CMC: coefficient of multiple correlation; SER: stacked ensemble regressor; KNN: k-nearest neighbors; WNN: wavelet neural network; FFNN: feed-forward neural network; ANN: artificial neural network. *—presents partial results of the studies. Bold text is used to distinguish the columns and rows.

## Data Availability

Data will be available when requested.

## Supplementary Materials

The following supporting information can be downloaded at: https://www.mdpi.com/article/10.3390/s26082502/s1, **PRISMA-ScR-Fillable-Checklist** [59].

## Abbreviations

MLP	Multi-Layer Perceptron
GRF	Ground Reaction Forces
IMU	Inertial Measurement Unit
SVM	Support Vector Machines
KNN	k-Nearest Neighbours
PCA	Principal Component Analysis
MeSH	Medical Subject Headings
LSTM	Long Short-Term Memory Neural Network
Bi-LSTM	Bidirectional Long Short-Term Memory Neural Network
CNN	Convolutional Neural Network
MAPE	Mean Absolute Percentage Error
FFNN	Feed-Forward Neural Network
WNN	Wavelet Neural Network
SFLR	Stepwise Forward Linear Regression
SVD	Singular Value Decomposition
SVR	Support Vector Regression
LASSO	Least Absolute Shrinkage and Selection Operator
LOSO	Leave-One-Subject-Out
MAE	Mean Absolute Error
MSE	Mean Square Error
RMSE	Root-Mean Square Error
rRMSE	Relative Root Mean Square Error
nRMSE	Normalized Root Mean Square Error
SPM	Statistical Parametric Mapping
AP	Anteroposterior Ground Reaction Force Component
ML	Mediolateral Ground Reaction Force Component
NN	Neural Network–Based Studies
LR	Loading Rate
pGRF	Peak Vertical Ground Reaction Force
A-PK	Active Peak
I-PK	Impact Peak
IMP	Vertical Impulse
CTT	Foot Contact Time
